# “Insta Residency:” Characteristics of Engagement With an Internal Medicine Residency Program Instagram Account

**DOI:** 10.7759/cureus.23565

**Published:** 2022-03-28

**Authors:** Thomas M Li, Danielle L Tepper, Alfred Burger, Matthew A Weissman

**Affiliations:** 1 Department of Medicine, Icahn School of Medicine at Mount Sinai, New York, USA; 2 Department of Medicine, Mount Sinai Beth Israel, New York, USA; 3 Departments of Internal Medicine and Pediatrics, Icahn School of Medicine at Mount Sinai, New York, USA

**Keywords:** graduate medical education, residency, social media, instagram, internal medicine

## Abstract

Background

Traditionally, medical training programs have been viewed and ranked according to factors such as national prestige, research opportunities and productivity, faculty prominence, and alumni success. While these components are still instrumental in attracting high-level applicants, the rise of social media has encouraged applicants to look beyond the traditional media put forth by institutions and turn to sources such as program-specific, departmental, or institutional social media pages for more rapid updates. To date, little has been written on how to maximize residency program social media use to engage target audiences of current residents, current faculty, other physicians, and applicants. To better understand how certain types of content lead to increased engagement, we analyze the Instagram social media platform of the Mount Sinai Beth Israel Internal Medicine Residency Program to identify potential trends in interactions to ultimately enhance the resident experience and institutional standing.

Methodology

We reviewed 257 posts on our Instagram account and calculated engagement of each category relative to the post count based on a method we developed. We used Instagram analytics to better understand our account’s reach.

Results

Posts highlighting social events had the highest engagement from the online community. Data also show that while the page is viewed by many people with the average medical student age, our page captures an even wider audience.

Conclusions

Instagram posts about the social element of our residency program generated statistically significant increased engagement. Institutions can use this strategy of focusing on the social aspects of the program to increase reputational scores within the medical and greater community.

## Introduction

Daily social media use as a source of education has increased dramatically in the 21st century. According to the annual report by the American media agency, We Are Social, the number of social media users grew by 13% in 2020 from 2019, bringing the global user count to nearly 4.2 billion [1]. Data indicate that social media users will spend almost two and a half hours on social media daily, with the total time spent on social media to 3.7 trillion hours for the year 2021.

As part of this growth, graduate medical education programs have turned to social media to build a community, attract applicants, and maintain academic prestige [2]. The academic uses of social media include using institutional websites and lecture recordings to circulate conference proceedings, promoting open faculty positions, and spurring trainee development [3]. Social networking sites (e.g., Facebook, Twitter, Instagram), blogs (e.g., Medium), and video sharing platforms (e.g., YouTube, TikTok) have further allowed cross-pollination of such interactions.

Previous work on social media in hospitals quantified faculty perception of its utility [4,5] and presence in various subspecialties [6-10]. Studies have also reported its utility in crowdsourcing ideas and cited a model using social media to justify academic promotion [11]. In 2020 and 2021, the coronavirus disease 2019 pandemic led to a halt in in-person interviews and social hours. Naaseh et al. highlighted these changes and conducted a small survey to investigate how people used social media during the 2020-2021 match cycle [12]. Heard et al. analyzed its use in urology [8] and noted that an increase in social media usage to convey the culture of the institutions that are missing during site visits was likely happening in many specialties. Many papers have reviewed the social media usage of individuals and programs during this period, particularly in surgical and procedure-heavy fields such as cosmetic dermatologic surgery [13], orthopedic surgery [14], and plastic surgery [15].

Despite the increasing reliance on social media during this difficult period, no study has identified how to increase social media interaction, particularly with residents and residency applicants and in Internal Medicine. Knowing what content best engages a particular target audience, residency applicants, for example, may contribute to increased yield and academic prestige.

In September 2018, the Mount Sinai Beth Israel Internal Medicine Residency created an Instagram account. The purpose of the account was to post photos and videos that provided updates about the program and illustrate camaraderie established in the wards, at social engagements, and during wellness events. A Twitter account was created at the same time to focus more on academic events and resident and faculty success.

In this study, the first aim is to analyze the content on the Mount Sinai Beth Israel Internal Medicine Residency Instagram account to determine whether certain types of content lead to increased engagement. The second aim is to assess the type of audience we reach. Understanding these trends may allow program leadership to strategize social media posts to recruit applicants and enhance the resident experience and institutional standing.

An abstract of this paper was presented in a virtual poster form at the Society for General Internal Medicine (SGIM) Mid-Atlantic Regional conference on October 29, 2021.

## Materials and methods

Instagram photos and videos were posted by Chief Residents during conferences in anticipation of specific events, or when they saw an opportunity to showcase the Program’s individuality. Chief Residents were not required to adhere to a posting schedule. We separated the first 257 Instagram posts on this account into seven categories: Fun Activities, Academic, Collegial Pictures, Resident Life, Faculty, Informational, and Other. For posts that fell into two categories, we chose the more prominent theme. Fun Activities were defined as non-clinical events; Academic included conferences or meetings; Collegial Pictures were defined as official group photos; Resident Life included photos taken during clinical experiences; Faculty were pictures of instructors or professors teaching; and Informational were posts highlighting data or advertising an event. To quantify engagement, we summed the number of likes a post received and the number of comments the post received multiplied by three. We included this adjustment to account for the greater weight a comment has in engagement, and this corrected sum was termed “points.” The sum of each category was calculated and divided by the number of posts in that group. Some in media have tried other methods to quantify these measures of engagement [12], but as no study gave more weight to comments, we developed our novel system. In summary, we defined engagement as the weighted sum of comments and likes on a given photo. Where necessary, one-way analysis of variance tests and Tukey post-hoc analyses were conducted using GraphPad Prism 9 to correct for the fact that the program had more social media followers as time progressed because later posts tended to have greater engagement than earlier posts. Statistical significance was denoted as p < 0.05. The project was reviewed by the Department of Medicine Quality Improvement Committee at Mount Sinai Beth Israel who deemed this as a quality improvement initiative not requiring a review by the Institutional Review Board.

## Results

Instagram is an accessible, online platform capable of attracting and maintaining social engagement

In the 18 months after the account’s creation, the Mount Sinai Beth Israel Internal Medicine Residency Instagram account gained 721 followers. There were 139 posts with an average of 45 engagement points per post. Twelve months later, the account had 1,229 followers and 257 posts with an average of 58 engagement points per post (Table 1). In these 12 months, content posted during this period received an average of 73 engagement points per post.

**Table 1:** Instagram account growth between March 2020 and May 2021.

	March 2020	May 2021
Followers	721	1,229
Posts	139	257
Engagement (Sum)	6,268	14,936
Average engagement/Post	45.09	58.34

Because the Instagram account is public, anyone with access to the internet can reach and interact with the content. Instagram Insight Analytics is a feature granted to certain business accounts on Instagram and grants a snapshot of an account’s activity over the past 30 days. Our Analytics revealed that our account reached nearly 1,400 unique accounts, garnered 1,275 content interactions, had nearly 48,000 impressions, 1,700 profile visits, and a 2% increase in follower count during this 30-day period. Instagram defines an impression as the number of times the page was shown to users; this differs from a profile visit, in which the user directly interacts with an account. Together, these data indicate that the Internal Medicine Residency Instagram account can attract and maintain an audience interested in our Program.

Posts highlighting social elements have increased engagement despite greater frequency

**Figure 1:**
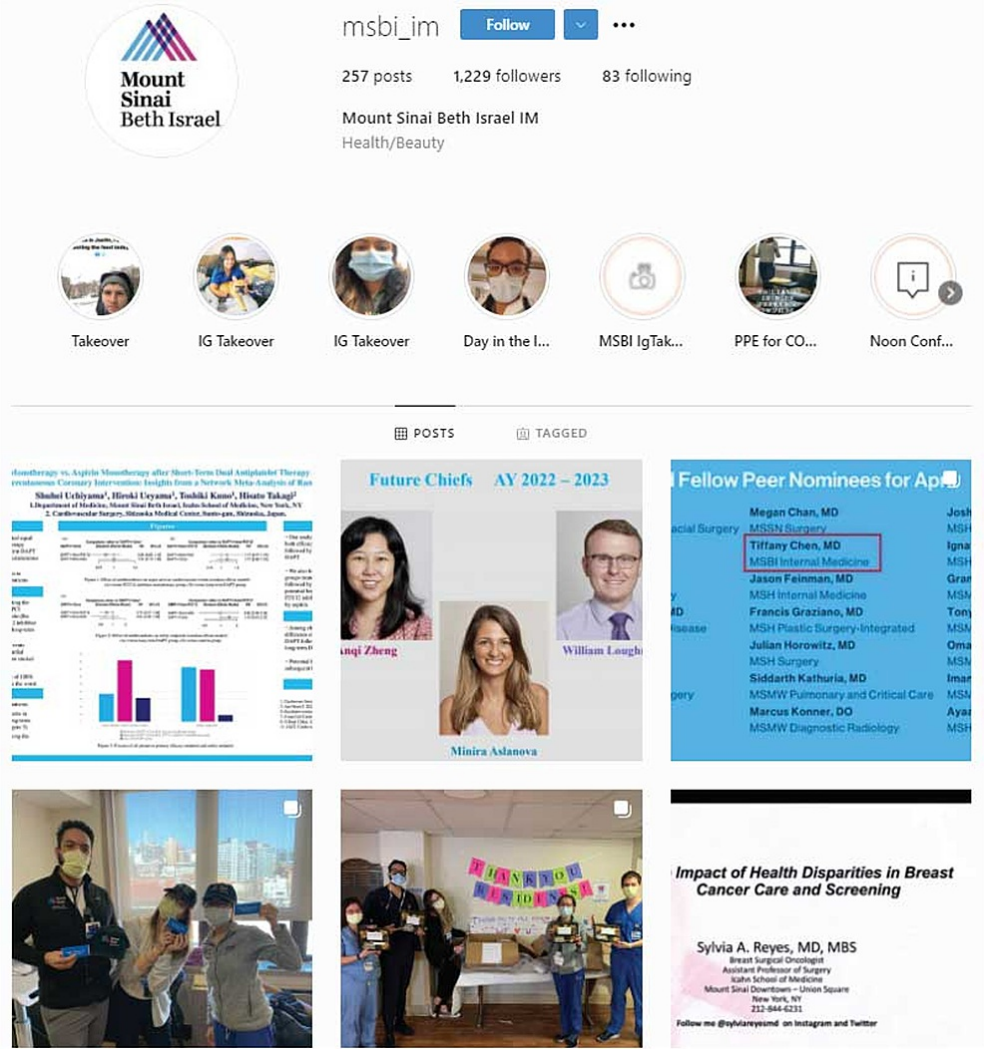
Screenshot of the Instagram account from an online browser.

The Instagram account contains various types of posts (Figure 1).

**Figure 2:**
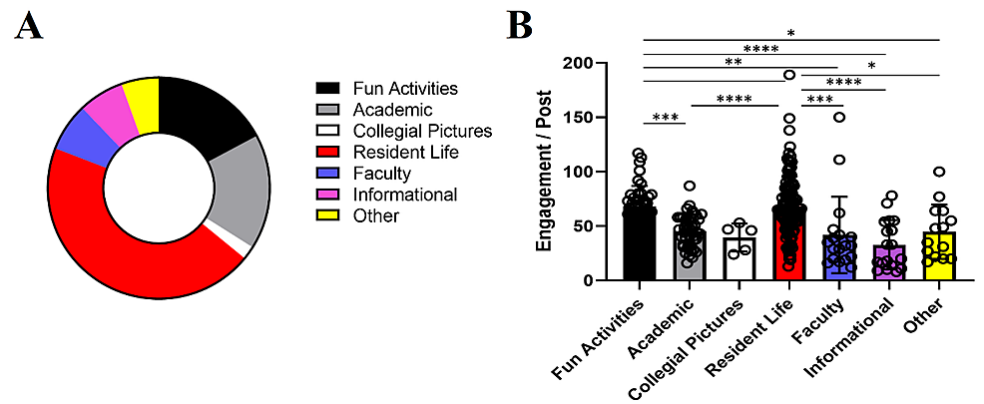
(A) Graph showing the distribution of content type of 257 posts from account creation to May 2021. (B) Normalized engagement per post utilizing an equation that gives comments greater weight than likes.

To ascertain if certain posts were more popular than others using engagement as a readout, we categorized each Instagram post into one of the seven categories described above. Resident Life content comprised a significant percentage of the account (44.9%), while Fun Activities and Academic posts made up 17.2% and 16.8%, respectively (Figure 2A). Posts about Fun Activities and Resident Life received an average of 68 points per post, which was significantly higher than engagement from other categories (Figure 2B). Our data indicate that posts on Instagram spotlighting resident life in and out of the hospital setting were more well-received.

Instagram mostly reaches resident-aged Instagram users

**Figure 3:**
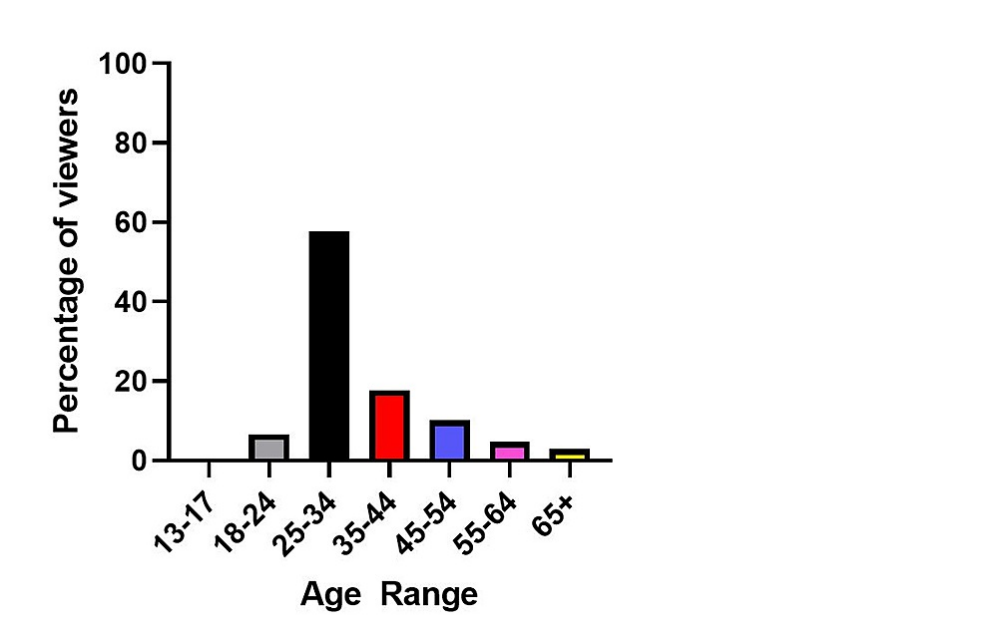
Age demographics of Instagram users who visited the Mount Sinai Beth Israel Internal Medicine Instagram page.

A secondary goal of the study was to determine the demographics of the Instagram account audience. To assess this, we looked at age ranges provided by Instagram Insight Analytics and saw that most users that interacted with our content were between ages 25-34 (57.8%) with the next bracket of ages 35-44 (17.8%) (Figure 3).

## Discussion

With social media becoming a large force in how institutions interact with individuals, we wanted to assess if our Program’s use of social media was efficient in reaching and engaging residents, faculty, and potential residency applicants. Furthermore, a new generation of applicants that has grown up with the internet is likely to turn to social media to supplement their understanding of programs and institutions. Studies delineating a role for social media increasingly demonstrate its value in both education and collaboration [6], and we wanted to better understand the characteristics of our Instagram account to address this.

Our Chief Residents did not have requirements to post specific types of content nor did they have a posting schedule. We recognize that a limitation of this study is analyzing one Instagram account from a single institution and the inability to definitively say who views our account despite showing gross age ranges. However, our unpublished data from a survey distributed to an incoming intern class in Internal Medicine show that Instagram is more popular than other social platforms. Knowing that applicants prefer Instagram over other social media tells leadership that strategizing Instagram posts to highlight social elements of the residency program may reach more applicants. Biannual, or even quarterly, reassessment of how users interact with our data may steer our Instagram content, allowing us to be contemporary and continue to increase program notability. By understanding what applicants, and ultimately residents, interact with online, our institution can enhance the resident experience by providing in-person social and academic events.

Future work can be directed toward understanding how social media influences residents and fellows during their application process and if targeting third- and fourth-year medical students is helpful in promoting engagement. Another avenue includes solely posting social posts for a month and comparing its cumulative engagement to a month where social posts were purposefully omitted. Other questions that arise from our study include: should Instagram and social media be used to appeal to applicants, engage current students, grow our patient population, solicit financial gifts, and hire new faculty? Moreover, how can institutions strategize posts?

## Conclusions

We found that Resident Life made up the majority of content posted on the Instagram account, and these photos garnered a higher level of engagement compared to Academics, Faculty, Informational, and Other posts. There was no difference in engagement between Resident Life and Fun Activities, suggesting that visitors to our page were looking for resident-based material. The majority of visitors to our account were of resident age. These results indicate that Instagram can be a valuable recruitment tool and, in particular, content posted about the residency program can engage a greater number of individuals.
